# Updating Darwin: Information and entropy drive the evolution of life

**DOI:** 10.12688/f1000research.10289.1

**Published:** 2016-12-01

**Authors:** Irun R. Cohen

**Affiliations:** 1 The Department of Immunology, The Weizmann Institute of Science, REhovot, Israel

**Keywords:** Evolution, Information, Complexity, Entropy, Cooperation, Death, Entropic Selection, Natural Selection

## Abstract

The evolution of species, according to Darwin, is driven by struggle – by competition between variant autonomous individuals for *survival of the fittest* and reproductive advantage; the outcome of this struggle for survival is *natural selection*. The Neo-Darwinians reframed natural selection in terms of DNA: inherited genotypes directly encode expressed phenotypes; a fit phenotype means a fit genotype – thus the evolution of species is the evolution of selfish, reproducing individual genotypes.

Four general characteristics of advanced forms of life are not easily explained by this Neo-Darwinian paradigm: 1) Dependence on cooperation rather than on struggle, manifested by the microbiome, ecosystems and altruism; 2) The pursuit of diversity rather than optimal fitness, manifested by sexual reproduction; 3) Life’s investment in programmed death, rather then in open-ended survival; and 4) The acceleration of complexity, despite its intrinsic fragility.

Here I discuss two mechanisms that can resolve these paradoxical features; both mechanisms arise from viewing life as the evolution of *information*. Information has two inevitable outcomes; it increases by autocatalyis and it is destroyed by entropy. On the one hand, the autocalalysis of information inexorably drives the evolution of complexity, irrespective of its fragility. On the other hand, only those strategic arrangements that accommodate the destructive forces of entropy survive – cooperation, diversification, and programmed death result from the entropic selection of evolving species. Physical principles of information and entropy thus fashion the evolution of life.

## Time for taking stock

Evolution of living organisms is probably the one incontrovertible “law” of biology, and Charles Darwin sponsored the idea ^1^. It only remained for biology to combine Darwin’s concept of *survival of the fittest* with Mendelian genetics and the discovery of DNA to generate what has been called the Neo-Darwinian synthesis – the ruling paradigm of today’s biology ^2^. After a half century of research, any ruling paradigm, however revered, needs reexamination in the light of the findings that have emerged since its conception. Science advances by periodic review of its most cherished teachings; an outdated paradigm is not mere excess baggage: it actually obstructs new ideas and new experiments ^3^.

## The Neo-Darwinian narrative

*Survival of the fittest* is the mechanism behind the *natural selection* that drives the evolution of life, as originally proposed by Darwin ^1^. The core idea is that replicating, autonomous living agents must compete for the necessarily limited resources provided by their environments. Consequently, living agents exist in a state of continuous struggle for survival – each agent fighting for its exclusive advantage. Since a phenotype is the expression of the individual’s genomic DNA, fitness is transmitted by the fittest individuals to their offspring. As a result, only those individuals most fit to their particular environments will win out, and the fittest genotypes will dominate and, by reproductive success, will eventually replace the less fit genotypes in the species. Succinctly said, the evolution of species is driven by individual selfishness. Living agents naturally shun death and strive to go on living; however, since death is inevitable, the payoff for fitness is success in generating fit offspring.

**Figure 1.**
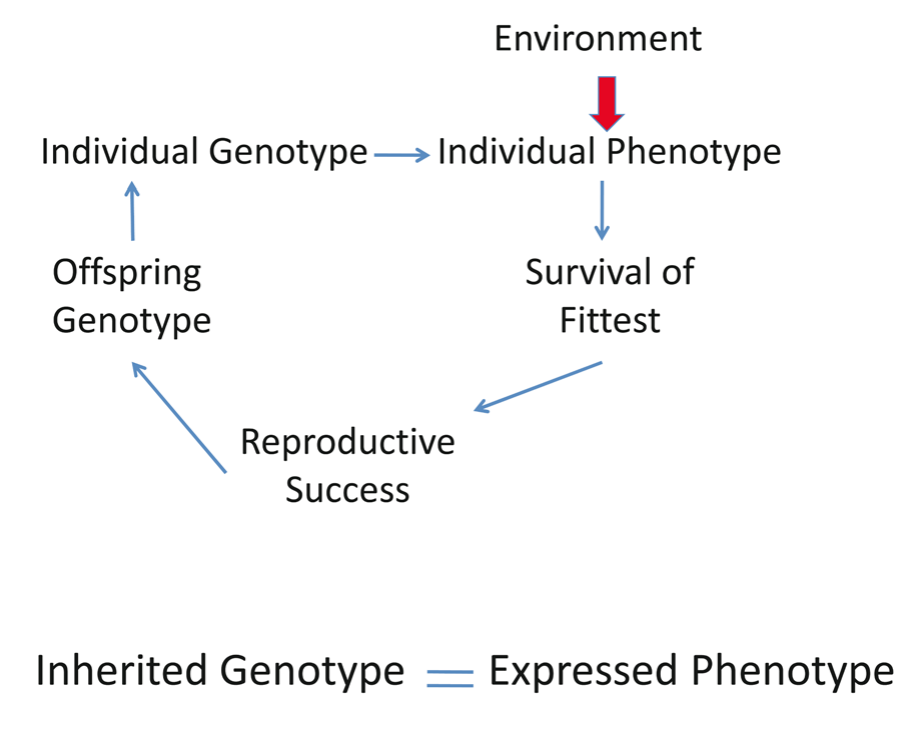
The Neo-Darwinian Paradigm. The seminal idea is that the inherited, *individual genotype* encodes the *individual phenotype* that undergoes fitness testing imposed by the environment. Organisms within a species differ in their genotypes/phenotypes and the individual with the fittest genotype/phenotype survives and, through reproductive success, enriches the species with the fittest genes. The process continues to cycle, continuously adapting the species to the changing environment.

With the discovery of DNA as both the organism’s hereditary endowment and the code that determines protein sequence, biologists, and the public at large, have come to envision DNA as the master program of life ^4^; the body and its functions are the overt expressions of one’s genotype; one’s inherited genome is one’s ultimate individuality. Hence, a fit individual who has passed on fit DNA to its offspring can be seen to compensate, at least conceptually, for the individual’s mortality; one’s genes residing in one’s offspring can persist despite one’s physical death. Successful breeding is not merely the payoff for fitness, it is life’s response to death. William Shakespeare, anticipating DNA, put it this way; “ *And nothing ‘gainst Time’s scythe can make defence save breed to brave him when he takes thee hence*”, Sonnet XII. Figure 1 summarizes the Neo-Darwinian understanding of evolution.

*Survival of the fittest* is often modeled using *game theory*: the struggle for dominance between competing agents is viewed as a *zero-sum* or *zero-determinant game* in which the winner prospers at the expense of the losers, and selfishness emerges as the most stable strategy of evolution ^5^. The selfishness of the individual phenotype is thus linked to the exclusivity of its formative genotype.

The game of life is assumed to reflect rational economics – the winning strategy is the one that is *optimally efficient*, under the circumstances, in exploiting time, energy and materials; evolution generates optimal genetic solutions – or so teach Neo-Darwinians.

## Shortcomings of the Neo-Darwinian narrative

**Figure 2.**
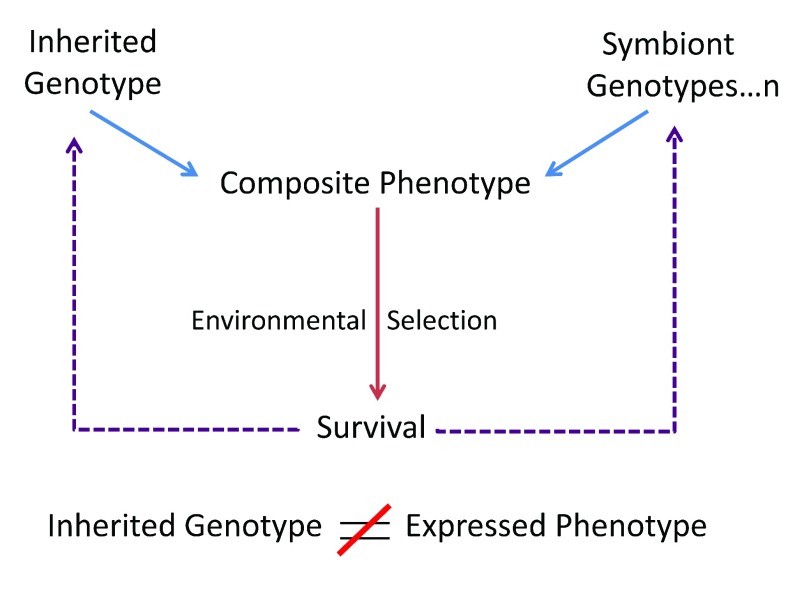
A multi-genomic, composite phenotype is tested for survival by the environment. Contrary to Neo-Darwinian teachings, the individual *phenotype* is not encoded exclusively by the inherited genotype. Rather, multiple genotypes generate the expressed, composite phenotype; the formative genotypes include the inherited genotype plus all the genotypes of the essential symbionts housed by the individual. The survival of the fit individual thus feeds back genetically both on the frequency of the heritable genetic alleles housed by the host species and on the frequency of the cooperating genotypes of the symbionts.

**Figure 3.**
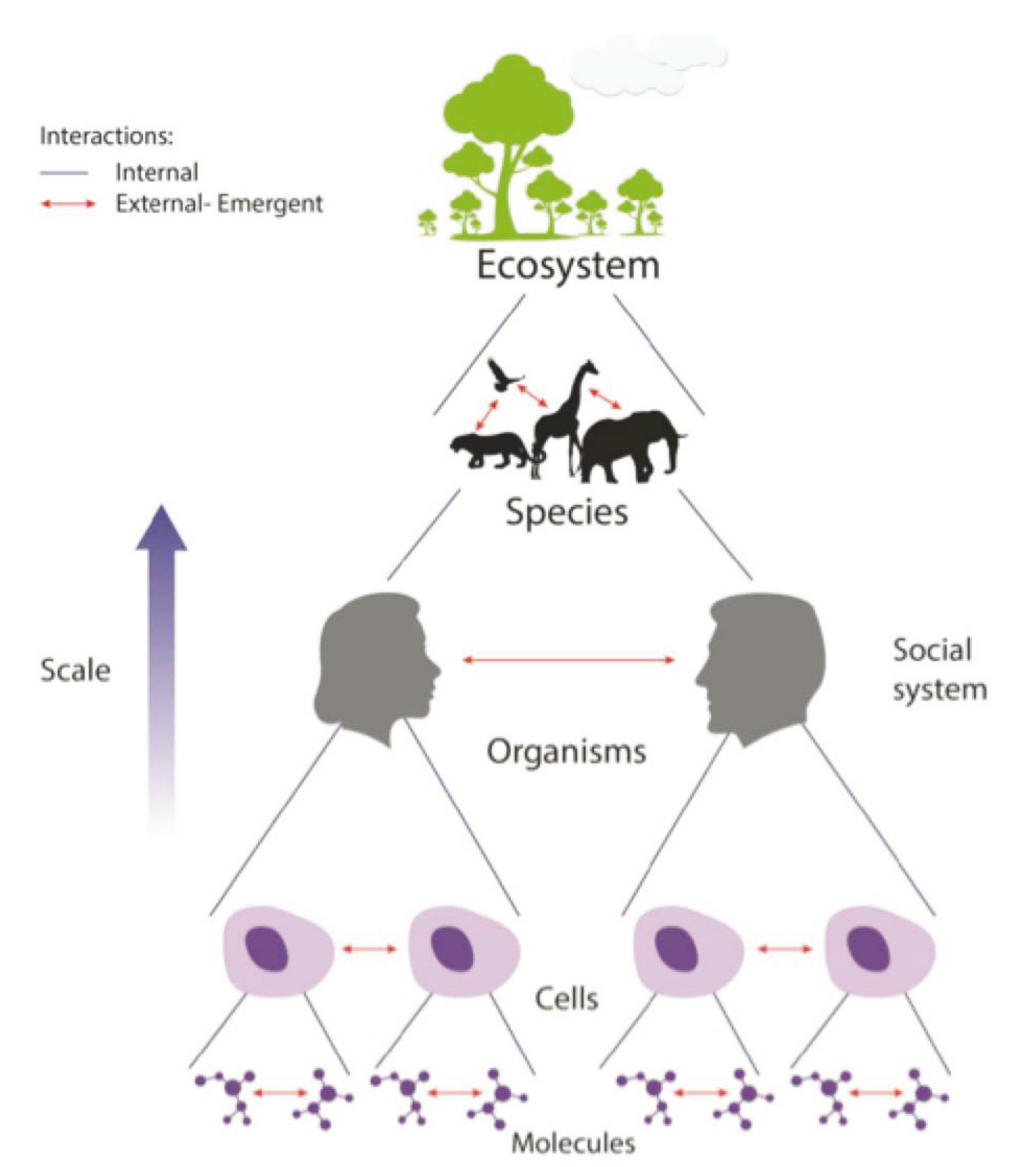
Life depends on nested, cooperative interactions, linked by eco-genes. A schematic view of multiple scales of life interacting within (internal) and between (external) living systems: interacting molecules form cells; interacting cells form organisms; interacting organisms form species; interacting species form ecosystems; and interacting ecosystems create the grand ecosystem that is the biosphere (not shown). The bonds between internally cooperating systems (red lines) and externally cooperating systems (dark blue lines) are encoded by eco-genes.

**Figure 4.**
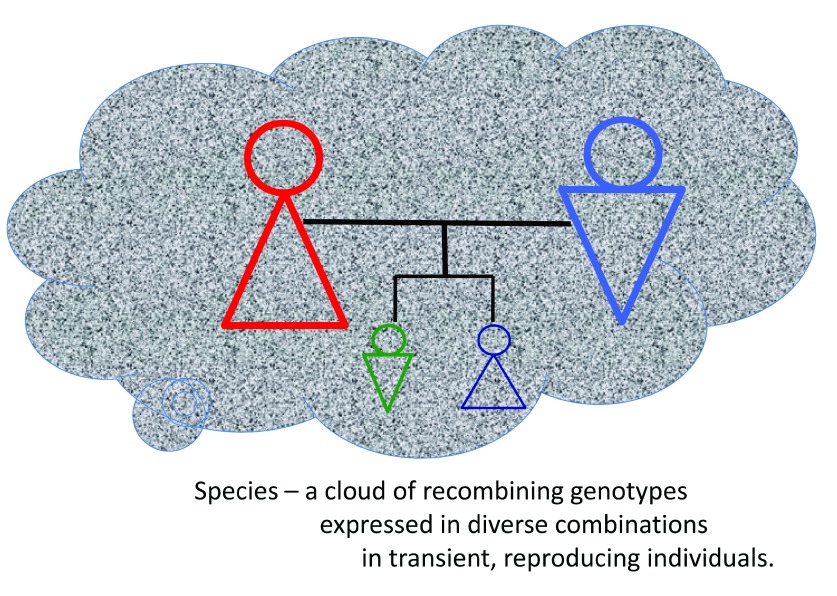
Sexual reproduction ensures continued diversification of heritable genotypes within a species. The cloud-like form represents a species and the stippled grey fill represents the frequency distribution of genes within the species. Two sexually reproducing individuals house genotypes that are each composed of a different combination of possible genes within the stippled fill of the species. The two offspring each bear their own species genotypes that are recombinations of parental genotypes. The individuals within the species are born, sexually reproduce and then die to maintain a frequency distribution of genes within the species that fits the life style and ecosystem arrangements that maintain the species.

More recent advances in the biomedical sciences have highlighted four universal characteristics of multi-cellular organisms that are not easily explained by selfish individual struggle and dominant reproduction:

1) *Mutual cooperation marks living systems.* A telling example of the importance of cooperative living – symbiosis – is the discovery that individual multi-cellular organisms, humans included, house as many bacterial cells – the microbiota – as eukaryote cells; indeed, healthy microbiota genes (the microbiome), which usually outnumber one’s inherited genes, are essential for health, and health is essential for adaptive survival ^6, 7^. Universal symbiosis contradicts the Neo-Darwinian assumption that the individual’s phenotype is the functional expression of the individual’s genotype; it is not. The living phenotype is actually a consortium that expresses the individual’s inherited genotype along with the genotypes of the individual’s symbionts – the individual phenotype emerges from cooperative interactions between multiple genotypes ( Figure 2); even identical twins house different symbionts, brains and immune systems. Hence, the individual is an ecosystem and the environment tests the fitness of the individual ecosystem, and not just the fitness of the individual’s inherited genes. Consequently, the survival and procreation of the composite individual feeds back on the frequency of particular genes in the breeding species and on the genes composing the symbiotic microbiome. In other words, even the basic individual is a group – the fittest individual expresses the fittest collective of interacting cells, prokaryote and eukaryote, within a single body; Neo-Darwinian *natural selection* now has to be considered as some type of *group selection* ^8^ Essential symbiosis obliges us to revise our understanding of the game plan of evolution ^5^.
  Beyond mortal individuals, life on earth is dependent on higher scale ecosystems involving networks of interacting species ( Figure 3). No species functions autonomously; a living system depends on supporting interactions within and between other systems ^9^: DNA, proteins, and other molecules exist thanks to network interactions within and outside of cells; cells cooperate with other cells to form organisms; even bacteria survive by social network interactions; organisms persist only through ongoing network interactions with other organisms, cells and molecules; species exist only within supporting ecosystems. Mutually supportive ecosystems characterize life on earth ( Figure 3). It is now clear that the evolution of life is the evolution of cooperation ^10^.
  A third example of the prevalence of unselfish cooperation is altruism – the sacrifice of one’s resources (even of one’s life) for the benefit of others. The very idea of altruism is clearly at odds with Darwin’s teaching that evolution is driven by selfishness. Neo-Darwinian explanations, however, can be derived, in principle, from positing some ultimate benefit to the self-sacrificing agent or to its genes, such as kin selection or other deferred advantages ^10^. Below, we shall see that cooperation arises more “naturally” when we consider a different force that molds evolution.
2) *Living systems invest in diversity and avoid uniform fitness by sexual reproduction*. A most obvious example of programed diversification is sexual reproduction. According to Neo-Darwinian thinking, fitness should reward the fittest with equally fit offspring ( Figure 1) – if not, what could be the meaning of reproductive fitness? On the contrary, sexual reproduction guarantees that your offspring will never replicate your exact fitness no matter how optimally fit you may be – sexual reproduction randomly mixes half your genes with the genes of another, whose fitness has not been tested by your ability to survive – your offspring could be more or less fit than you ( Figure 4). Indeed, a mutation encoding parthenogenesis should replace sexual reproduction by its efficiency alone ^11^. Some have attempted to explain sexual reproduction as insurance against rapid shifts in the environment that require a shift in the optimal genome; sexual reproduction, nevertheless, remains an unsolved paradox for the Neo-Darwinian worldview ^12^.
  Sexual reproduction is not the only example of the evolution of less than optimal fitness. Indeed, the *handicap principle* proposes that exaggerated or burdensome overt traits serve as signals to perspective mates that prove the selective value of the suitor’s other, more covert evolutionary traits ^13^; the cumbersome tail of the peacock assures the peahen that he must bear very fit genes to have escaped predators till now; here, a covert advantage (strength) is seemingly advertised by a wasteful or dangerous overt display (the ungainly tail). Mass migrations of salmon across oceans ^14^ and migrations of lemmings to their deaths ^15^ do not seem very economical. The reader can supply his or her own favorite example of the irrationality of evolution in devising less than optimal creatures and illogical behaviors.
3) *The paradox of organized death:* The relentless struggle for survival envisioned by Darwin is challenged by the fact, long ignored, that much of the internal molecular machinery of the cell – genes included – is devoted to multiple processes of self-inflicted death by apoptosis and other means ^16^. Organisms cannot develop and survive unless large numbers of their constituent cells commit suicide or are killed at regular intervals and under specific circumstances. Cells with irreparable DNA damage kill themselves for the good of the individual; in many social species, weak males refrain from reproduction, and altruistic humans even sacrifice themselves, for the good of the species.
4) *The paradox of accumulating complexity:* An obvious feature of evolution has been the emergence of increasing complexity – from prokaryote to human culture. There is yet no accepted definition of complexity, but however you choose to define it, if humans are not more complex than are bacteria, there is something wrong with your definition of complexity. Likewise, however you choose to define fitness, if bacteria are not more fit than are humans, then there is something wrong with your definition of fitness. Bacteria have never undergone any of the mass extinctions that have visited other more complex forms of life; indeed, the more complex creature is the more fragile creature ^17^. Who will better survive global warming, the rise of the oceans, and the destruction of biodiversity – we mammals or the bacteria? If survival of the fittest is the driving force of evolution and its measure of success, why did evolution not stop with bacteria? How could *survival of the fittest* drive evolution to fashion and maintain more complex, but more fragile organisms? Darwinian explanations for the evolution of complexity have been explored in detail ^18^. But let us consider a non-Darwinian explanation for the evolution of complexity, cooperation, sex and programmed death.

## The evolution of complex information and its adaptation by entropic selection

Since Darwin’s discovery of evolution, we have succeeded in characterizing the molecules, cells and processes that form organisms and species. We now know that living systems owe their existence to particular arrangements of their internal component parts and to the dynamic interactions between them ( Figure 3): cells are specific arrangements of interacting molecules; organisms are specific arrangements of interacting molecules and cells; species are interacting arrangements of organisms; ecosystems are interacting arrangements of species ^9^. In each case, the details of what is arranged and what interacts are the domains of different branches of enquiry and experimentation. Nevertheless, we can adopt the formulation of Claude Shannon and define the common feature of any specific arrangement of any matter or process as *information* ^17, 19^. Information, according to Shannon, can be measured as the degree to which the particular arrangement of interest differs from a random collection of the same component parts. In other words, irrespective of whether we are dealing with carbon atoms in a macromolecule, molecules in a cell, cells in an organism, organisms in a species or species in an ecosystem – whatever manifests nonrandom arrangement bears *information*; information by this definition is not limited to words in a message. Information in the narrow sense of arrangement is distinct from information expressed as *knowledge* or *meaning*; knowledge and meaning express *applications* of information – they are ways arrangements can be used to achieve goals or support interactions ^17^. Nevertheless, information, defined *as arrangement per se,* serves as a necessary substrate for the creation of knowledge and meaning. Since the essence of living systems at all scales is their particular arrangements of internal components and their internal and external interactions, we can say that living systems are formed by essential information, irrespective of just what materials or actions are arranged ^17, 20^.

This sounds uselessly abstract; immunologists, for example, are interested in lymphocytes, neurologists in neurons, ecologists in food chains, among other specifics – each field of biology strives to elucidate the details comprising a domain of study; what is to be gained by lumping diverse living systems together as forms of *information*? The gain is that this narrow definition of information provides a unifying concept for analyzing evolution applicable to all its varied manifestations. In our bird’s eye view of evolution as a seminal process, we are not interested in the fine details of this or that gene, protein, organism, society or ecosystem; we want to see the big picture in one encompassing perception. As we zoom out, we see that the strategic path of evolution can be heuristically reduced to a reconciliation between the two opposing fates of information: its amplification and its destruction. The instantiation of these fundamental forces of nature shape the large-scale strategy of evolution.

## Information is autocatalytic and generates complexity automatically

Henri Atlan has formalized the idea that information in any system will increase automatically when two conditions are fulfilled: the replication of existing information into multiple copies and some variation in at least one of the replicates ^21, 22^. A detailed presentation of Atlan’s formulation is beyond our present scope, but empirically we know, for example, that evolution has been marked by processes of gene duplication and variation ^23^ – Atlan’s two conditions for increasing complexity. Also note that evolution is associated with pleiotropy – any gene, molecule, cell, or organism almost always performs more than a single function ^24^. Indeed, any living system engages in more than one network of interactions at different stages in its life history. Provided there exist a threshold concentration and flow of compatible arrangements, a given arrangement, such as a molecule or cell, is likely to roam into additional networks of interaction during development, maturation, or aging. In other words, information will tend to have unforeseen side effects ^25^. Complexity increases because the more information there is, the more likely that that information will become available for new engagements and, hence, for increased complexity and pleiotropism.

The automatic growth of complexity can be seen in the evolution of prokaryote cells into eukaryote cells: Eukaryote cells are demonstrably more complex than are prokaryote cells because the eukaryotes feature nuclei ( *eu* – true; *kary* – nucleus) and other complex organelles and more complicated organizations of DNA (introns and exons, for example). It is now generally accepted that the first eukaryote emerged from endosymbiosis, the amalgamation of several prokaryotes into a single unit cell ^26, 27^. What made a group of prokaryotes enter into a consortium to form a new, more complex cell type? Nobody really knows, but let me propose that once there emerged a sufficiently high concentration of different prokaryote types hanging out in the same neighborhood, sooner or later some of them, by chance, were likely to fuse their cell walls or otherwise ingest one another to form a more complex cell. The amalgamation happened by chance, but why did it take about a billion years to evolve a viable prokaryote? To my mind, prokaryote amalgamations were probably forming spontaneously and breaking up spontaneously all the time, but it took a billion years to obtain, again by chance, just the right combination of disparate prokaryote elements needed for the survival and replication of the seminal progenitor eukaryote. A threshold of complex information will always recombine and vary to generate even more complexity. Growing complexity, however, is sculpted by *entropic selection* – the complex arrangements that flourish are those that survive the trimming imposed by entropy – the inevitable and relentless destruction of information.

## Entropic selection channels the outcome of evolving complexity

Entropy, like Shannon-type information, has a formal, computational definition – entropy is a fundamental concept in thermodynamics ^28^. Actually, information and entropy are sister concepts; Shannon formulated his concept of information using Boltzmann’s theorem for entropy ^19^: The measure of information in a particular arrangement is relative to its improbability as opposed to the likelihood of its randomness; the influence of entropy on an arrangement is relative to the probability of its random disorder.

But in the present context we only need note that entropy ensures that specific arrangements ultimately fall apart spontaneously; the more complex the arrangement, the greater its fragility and the more likely it is to disintegrate. Even the optimal solution in the end will fail. Therefore, the complexities that emerge during the evolution of living systems will be selected by their ability to forestall, circumvent, or persist in the face of their inevitable destruction wrought by entropy – the outcomes of evolution are thus channeled by what we shall call *entropic selection*.

Note that Darwin’s *natural selection* takes place whenever variant individuals compete for survival in a resource-limited environment. Struggle is essential; *natural selection* will not operate in the absence of competition. *Entropic selection*, in contrast, operates wherever entropy operates, even in the absence of variant individuals or environmental straits. And entropy operates everywhere. In other words, principles of physics – including information and entropy – sculpt the evolution of life. Let’s see how this informational-entropic view of evolution accounts for strategic characteristics of living systems that are not easily explained by the Neo-Darwinian paradigm:

1. *The prevalence of cooperative interactions:* Entities that are involved in ongoing cooperative interactions are less likely to fall apart than are the same entities in a state of isolation or inactivity. Theoretical explanations exist but need not be invoked; the fact is readily observable: a house tends to fall apart unless somebody lives in it; enforced bed rest can quickly dispatch a previously active, apparently healthy old person; retirement can be deadly; active couples live longer than do lonely people; growing cells have a longer half-life time than do static cell cultures, and so on. Mutual interactions sustain the interacters and delay their dissolution. Selfishness is not viable; entropic selection ensures stable, natural cooperation.
  Our symbiosis with our gut microbiota exemplify advantageous mutual cooperation; we house and feed the bacteria and they, in turn, help us digest foodstuffs, provide needed vitamins and other metabolic products, prime the development of our guts and immune systems ^7^, and possibly of our brains ^29^. Altruism is good for bonding lovers, families, tribes, and societies; strong societies, in turn, are good for individual achievement, and especially for those in need such as orphans, the aged, and the debilitated. Finally, mutual cooperation between different species is essential for the ecosystems that maintain all life. The living world provides many more examples of cooperation than it does of selfish struggle ^10^; we can thank entropic selection for that – we’ll return to the function of struggle below.
2. *The prevalence of sexual reproduction and the organization of species:* Sexual reproduction guarantees genome reshuffling ^11, 12^, and so prevents any single dominant genome from taking over the species. Continuous diversification of individuals allows the species to survive the ultimate failure of its “most successful” individually expressed genotypes. Sexual reproduction is not merely Neo-Darwinian insurance against a possible future need for a new optimal genome; sex-mediated gene reshuffling is a sound response to entropic selection. The greater the variation within and between interacting systems, the greater the likelihood of survival – a diverse system manifests flexibility and resilience because it is not limited by a single essential set of components or a fixed plan of action. Entropic selection rewards diversity; Darwinian survival of the fittest in contrast, should reduce diversity by imposing a uniformly optimal fitness.
  Entropic selection also explains why life evolves as species – a species is a collective of arrangements that express a frequency distribution of alternative genomes and diverse interaction pathways ( Figure 4). The individual members of the species are each particular instantiations of alternative collections of recombining genes and interaction networks; the failure of one or more individual alternatives does not wipe out the species. Thus, a species is materialized by its individual members who breed to maintain a frequency of alternative genotypes that fit the life style of the species in its environment. Successful species are composed of alternative individuals, not of optimal or master programs ^30^. Perhaps there are no master programs around today because species dependent on single master programs were extinguished along the way; a species with only one or a few dominating genotypes, for example, would go extinct as soon as entropy inevitably would destroy that master genotype.
3. *Programed death and turnover:* Organized death ^16^ and regulated turnover of molecules, cells and individuals enable species to resist their dissolution by entropy, which kills by irregular debility or accident; programmed death is more tolerable because it kills at appointed times, locations and states. Organized, regulated self-destruction, followed by rebirth, is a strategy for surviving entropic selection – systematic *shut down* and *restart* of its component parts keeps an individual body alive for a lifetime and a collective species extent for an eon ^17, 24^. The characteristic life spans of individuals have evolved to suit the life style of the species, be it 3 days or 100 years or, in the case of certain trees, a thousand years.
4. *The growth of complexity:* Accumulating complexity, as we discussed above, can readily be explained by the autocatalysis of information, ^21, 22, 24^; Complexity, intrinsically fragile and contingent, is certainly not an outcome of *survival of the fittest*; complexity, despite its fragility, is inherent in the natural history of information – the emergence of complexity is inexorable.

## The evolutionary role of competition

We have made the point that individual struggle and survival of the fittest do not easily explain many of the large-scale strategies of evolution. Nevertheless, competitive struggle is a fact of life so prominent that Western Society, in the wake of Darwin, continues to see competition as the driving force of evolution. So what might be the functions of competition and struggle in evolution driven by entropic selection?

The outcome of struggle depends on whether or not it results from co-evolution of the antagonists; co-evolved competition is characterized by an interaction that should be beneficial for both parties. For example, individual zebras are killed by lions, but such co-evolved predator-prey struggles support the health of both antagonists at the scale of species ^31^; individuals are destined to die in any circumstance, but species of predators and prey thrive on organized individual predation. The monopoly on reproduction enjoyed by alpha males and females within a breeding population maintains the frequency of useful genes and establishes a functional social order within the species. Immune interactions with frequently encountered infectious agents are usually good for both host and parasite ^24^. Social systems do better with open competition for positions of responsibility. Life at the scale of species has evolved to benefit from certain organized individual struggles. Struggle between co-evolved partners serves the adaptation of living species to entropic selection; co-evolved competition is just a different face of cooperation.

In contrast to co-evolved species competition, novel struggles between unaccustomed antagonists may destroy existing ecosystems and lead to a loss of biodiversity. The prehistoric migration, for example, of human hunters from Asia into the Americas led to the extinction of many species of animals ^32^; the newly arrived human predators eradicated prey species that had previously supported other predators – many of the co-evolved predators and prey species became extinct. More recently, we have seen ecosystems destroyed by invading plant or animal species ^33^. We can conclude that co-evolved competition is likely to benefit the antagonistic species, while new struggles may damage previously operating ecosystems – until a new state of equilibrium can emerge; some wolves, for example, have co-evolved with human culture to generate dogs and cynophiles ^34^. Competition, in summary, has more than one role in evolution; it can help life resist entropic selection, but it can also act as an agent of disorderly destructive entropy, which, paradoxically, may engender from time to time new ecosystems.

## Entropic selection and natural selection compared

Table 1 outlines the major distinctions between Neo-Darwinian natural selection and entropic selection. Neo-Darwinians see the genome as *life’s program*; the advance of evolution is *driven* by competition between diverse autonomous agents, each struggling for survival and successful procreation; the unit *vehicle* of evolution is the individual, autonomous agent; the evolutionary *strategy* is to win a zero-sum game in which the most fit agent in the particular environment survives at the expense of the less fit – survival of the fittest; the *reward* is the reproductive success of the fittest agents leading to enrichment of the species with optimal genes most suited to the environment ( Figure 1); a new, divergent species can emerge in a new environment.

**Table 1.** Two views of evolution: Natural selection & entropic selection.

	Natural selection of autonomous agents	Entropic selection of informational systems
Life’s program:	The genome	Network interactions
Driving force of evolution:	Competition between agents	Growth of information and its dissipation by entropy
Game plan of evolution:	Zero-sum game between competitive antagonists in accommodating to the environment; *Survival-of-fittest* individual	Accommodating to entropy & to the environment: Win-win game/Cooperation; Diversity/Gene reshuffling; Organized death & restart; *Survival-of-fitted* species
Vehicle of evolution:	The individual	The species
Outcome:	Optimal individuals dominate the species; New species arise in new environments	Increasing complexity; Networks of species fashion their environments

**Figure 5.**
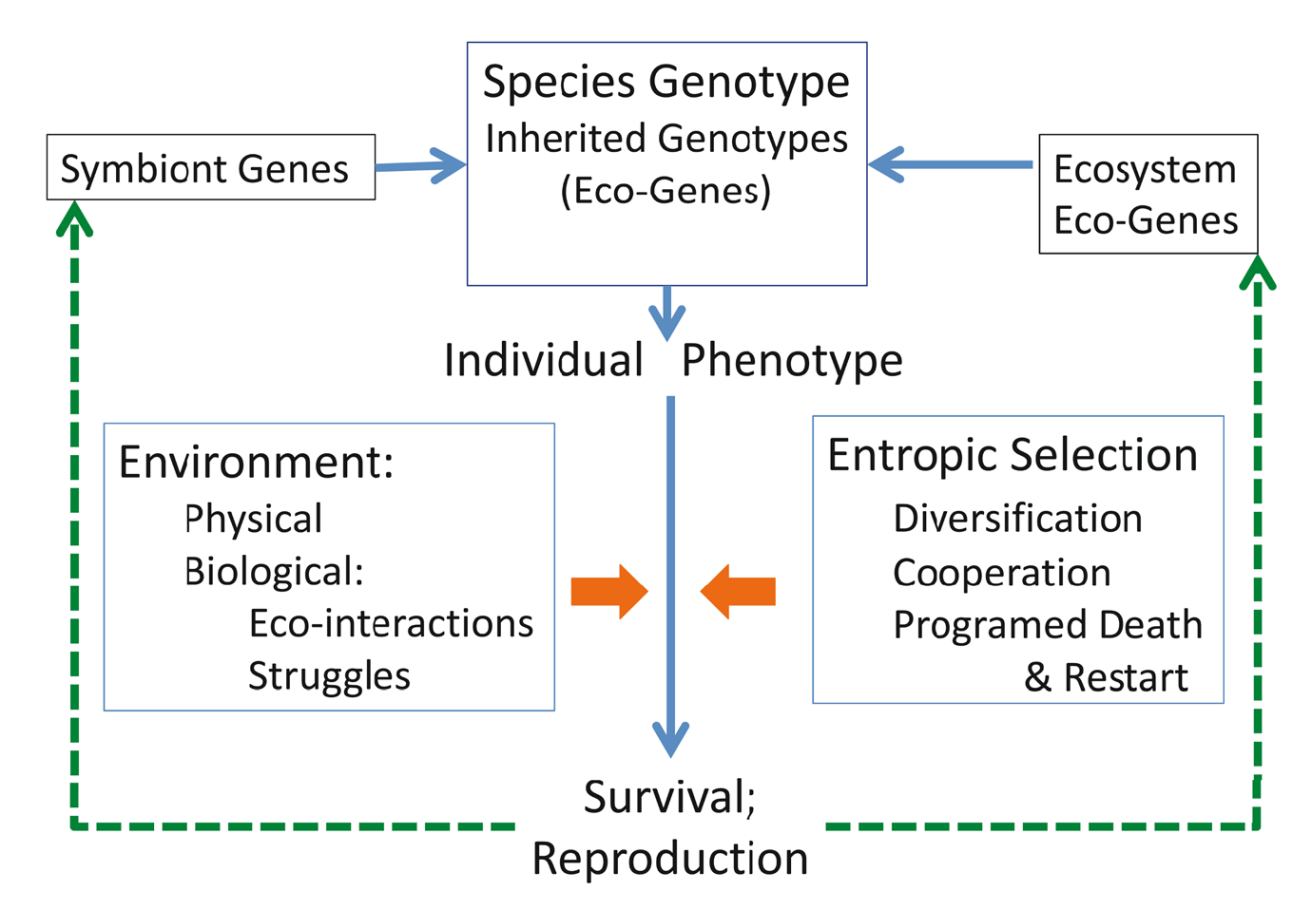
The entropic selection paradigm – a schematic summary. An individual phenotype of a multi-cellular species expresses a composite genotype that emerges from three cooperating sources: a subset of genes inherited from the species (including eco-genes); genes borne and expressed by its symbionts; and eco-genes expressed by its ecosystem partners. The individual is then tested by its ability survive in its environment – both the physical (temperature, oxygen tension, pH, etc) and the biological (eco-interactions suitable for sustenance, reproduction, etc). In parallel, entropic selection tests whether the individual manifests the requisite diversification, ability to cooperate, and programed death and restart that suit the life style of the species. Successful individual survival and reproduction feeds back on the distributions of genes in three compartments: in the species, in the symbionts and in the ecosystem partners.

Entropic selection, in contrast, sees living systems as the embodiment of information; evolution is thus driven by the two inevitable fates of information – amplification by autocatalysis and dissolution by entropy. *Life’s program* ( Figure 3) emerges from enmeshed, mutually supportive networks ^30^; the unit *vehicle* of evolution is the species – a continuously recombining population of alternative genotypes ( Figure 4); the *strategy* of living systems is to accommodate entropic dissolution by evolving supportive cooperation within networks of ecosystems in a win-win game, which is played by continuous diversification through sexual reproduction and other means, and by organized death and restart to ensure survival of the fitted; the *outcome* is the evolution of increasing complexity in which the environment itself is fashioned by enmeshed, interacting biological networks ^35^. Figure 5 summarizes the process.

## The mechanism of entropic selection

Entropic selection can account for the strategic features of evolution discussed here; but how does this selection actually operate? Ultimately, the effects of entropic selection must be based on the actions of genes; genes, after all, are the bearers of heredity. Sexual reproduction ensures continuous diversification of genotypes within species, but what genes encode the mutual cooperation inherent in symbiosis and in ecosystems? Moreover, how does entropic selection prevent the emergence of selfish genes ^36^?

Selfish genes that sabotage cooperation are destined to undergo negative entropic selection because cooperation is an essential element in preserving living systems; systems that try to stand alone fall. To counteract the emergence of selfishness, the gene pools of co-evolved species must also include genes that positively encode cooperation, including cooperative competition. Such cooperation-enhancing genes enable each system to identify and interact with signals specific for the collaboration. Let’s call these genes eco-genes (from Greek *oikos*, one’s house). Eco-genes are not a theoretical invention; existing eco-genes are already known:

The guts and the gut immune systems of mammals, for example, are equipped with receptors that accommodate symbiotic microbiota and distinguish them from microbial pathogens ^37^; indeed, the tolerance of the immune system towards symbiotic microbiota highlights the impossibility of defining an immutable “immune self” – the “self” is an interactive process rather than a closed entity ^24, 38^; in fact, the individual immune system is an ecosystem of interacting cells ^39^. Struggles between predators and prey are organized by sight, smell and taste receptors that mark what should be hunted and eaten by the predators and avoided by the prey: mice and cats, for example, innately recognize one another; zebras and lions signal each other clearly to establish a stable equilibrium between hunters and hunted ^40^. Humans are born with visual systems that recognize faces – face recognition serves as a foundation for human bonding and social interactions ^24^. The concept of eco-genes, in short, includes all the genes involved in essential interactions between individuals and between species that organize life in the face of entropic selection; particular eco-genes are too numerous to enumerate here, but their expressions operate to hold together species and ecosystems.

Beyond the process of evolution, the concept of *survival of the fittest* fosters a mindset whose consequences impact human social, economic and political behaviors. In contrast to Darwin’s *natural selection* by selfish competition, I have argued here that we can explain evolution in a more factual way as the evolution of interacting *information* and its pruning by *entropic selection*. This view founds biology on fundamental physical laws, and attributes the evolution of life to the dance of creation and destruction. Best of all, it teaches us that cooperation is a saving grace; fitness is fittedness.
