# Supplementary material for: Updating Darwin: Information and entropy drive the evolution of life
Source: F1000Res. 2016 Dec 1;5:2808. [Version 1] doi: 10.12688/f1000research.10289.1 (PMC5200945; doi:10.12688/f1000research.10289.1)
Supplement: Peer review history

# Peer review: Referee response for version 1

**DOI:** 10.5256/f1000research.11081.r18271

**Published:** 2016-12-28

**Authors:** Daniel M. Altmann

**Affiliations:** 1 Division of Immunology and Inflammation, Department of Medicine, Imperial College London, London , UK

In this piece Cohen gives a masterclass to young scientists that our aim is, not just to accrue datasets, but to ponder, synthesise and then write something thought-provoking, challenging, elegant and readable. Here he considers a timely update/iteration to the Neo-Darwinian paradigm, proposing a model of evolution of interacting information, with pruning by entropic selection. 

 The title and abstract are appropriate.

 His case is elegantly made, drawing widely on biological evidence and models to construct the argument.

 One might perhaps have wished in one or two areas for more detailed exposition of recent data in the light of his hypothesis: while interaction of the microbiota with host immunity is briefly alluded to, this has been one of the fastest growing areas in the past few years and surely offers food for thought. Also, any Neo-Darwinian reappraisal surely needs to incorporate space for evidence from environmentally-determined epigenetic modifications of the genome and impacts thereof on survival?

I have read this submission. I believe that I have an appropriate level of expertise to confirm that it is of an acceptable scientific standard.

# Peer review: Referee response for version 1

**DOI:** 10.5256/f1000research.11081.r18297

**Published:** 2016-12-07

**Authors:** Eugene Rosenberg

**Affiliations:** 1 Department of Molecular Microbiology and Biotechnology, Tel Aviv University (TAU), Tel Aviv, Israel

This is a well-written, thought-provoking article that challenges the foundations of Neo-Darwinism and presents an alternative concept for the evolution of complexity. To begin with, Cohen argues that Neo-Darwinism fails to explain cooperation, biological diversity, programmed death and complexity, which we now know are all hallmarks of life on this planet. Although these phenomena appear to contradict the selfishness of Darwinian evolution, evolutionary biologists have attempted to reconcile them with existing theory, more or less successfully. The co-discoverer of evolution, Alfred R. Wallace, argued that a characteristic can evolve also because it benefits the group even though it may be harmful to the individual possessing it. Subsequently, evolutionary biologists referred to this latter concept as group selection. Recently, a number of prominent evolutionary theorists have come to see the body of ideas known loosely as "multi-level selection theory" as a potent explanatory principle. Selection can operate simultaneously at different levels of the biological hierarchy, including groups (cooperation).

 The most interesting and important part of the article is the concept of viewing life as the evolution of information. Information can increase by autocatalysis and be destroyed by entropy. According to Cohen, physical principles of information and entropy drive the evolution of life. Entropic selection operates wherever entropy operates, even in the absence of variant individuals, environmental constraints or competition. This is a novel concept.

 The paradigm-changing concept presented in this article raises many questions that should be addressed by scientists from different disciplines. How exactly does information and entropy drive complexity at the cell and molecular levels? How does enthalpy enter the picture? Most important, how can this novel idea be tested? Hopefully, this article will inspire an in-depth examination of Neo-Darwinism.

I have read this submission. I believe that I have an appropriate level of expertise to confirm that it is of an acceptable scientific standard.
